# Sleeve gastrectomy *versus* mini-gastric bypass and their effects on type II diabetes mellitus and weight loss outcome

**DOI:** 10.25122/jml-2021-0155

**Published:** 2021

**Authors:** Mohammed Abd-Zaid Akool, Samer Makki Mohamed Al-Hakkak, Alaa Abood Al-Wadees, Ashraf Sami Muhammad, Safauldeen salim Al Baaj

**Affiliations:** 1. Department of Surgery, Faculty of Medicine, Jabir Ibn Hayyan Medical University, Najaf City, Iraq; 2. Department of Surgery, College of Medicine, Kufa University, Najaf City, Iraq

**Keywords:** sleeve laparoscopic gastrectomy, laparoscopic mini-gastric bypass, BMI, diabetes mellitus type 2, HbA1c

## Abstract

Bariatric surgeries such as sleeve gastrectomy; mini-gastric bypass surgery are successful weight reduction surgeries which significantly impact metabolic syndrome. The purpose of this research was to assess the impact of laparoscopy gastrectomy and mini-gastric bypasses on weight decrease and diabetes remission of diabetic mellitus type 2 through two years of monitoring. Furthermore, this study looked at the difference between the two procedures regarding their efficacy and identify which one is proper for patients according to their comorbidities. A prospective study was held in Al Sadder Medical City and Al-Gadeer private hospitals in Al-Najaf city, Iraq, from January 2016 to February 2018. The study included 35 obese and morbidly obese patients with a known history of diabetes mellitus type 2, diagnosed from at least two years before surgery. 15 patients undergo uneventful laparoscopic sleeve gastrectomy (6 females and 9 males). 20 patients underwent uneventful laparoscopic gastric mini bypass surgery (6 females and 14 males). In addition, the patients were followed in the short-term postoperatively (3, 6, 12, 24 months) by monitoring their BMI, weight loss, and HbA1c. There was a decrease in BMI of about 45% from the baseline BMI in sleeve gastrectomy surgery and a decrease in HbA1c of about 45%, less than 6%. In gastric mini-bypass surgery, there was a decrease in BMI of about 47% from the baseline BMI and a decrease in HbA1c of about 45% from the baseline less than 6%, during a 24-month monitoring. Both surgeries were fruitful and had efficient results on patients, but the gastric mini bypass was more efficient than sleeve gastrectomy in controlling and remission of DM type 2 without the need for medications. A long-term study should be performed to reveal their effect and benefits to the patients.

## Introduction

Morbid obesity causes serious public health problems worldwide, and the prevalence has risen repeatedly in Asian countries in recent decades [1]. Obesity surgery is the most efficient management for such patients. The main purpose of obesity surgery (BS) is to decrease weight of the body or body mass index (BMI). However, there is increasing recognition that surgery can affect many health comorbidities accompanying obesity, like diabetes mellitus type 2 (DMT2), uncontrolled lipidemia, and sleep apnea. Surgery for obesity was initially recommended by the National Institutes of Health (NIH) in 1991. Over the last few decades, numerous worldwide societies have adapted. BMI thresholds and comorbidities are widely used in defining bariatric surgical indications [2]. The criteria of WHO recorded that the BMI thresholds for obesity in Asian people are less than in the western population as health risks associated with obesity have a tendency to happen at a lower BMI threshold. Globally, the number of obesity surgeries has significantly increased since laparoscopy techniques were introduced, which are efficient, safe, and substitutes to open approaches. Laparoscopic gastrectomy (LSG) is now a common weight reduction procedure globally, with a growing prevalence over the last ten years [3]. In 2013, LSG was the second most common weight reduction surgery globally (37%), followed by gastric bypass Roux-en-Y (RYGB), with a significant increase from 2003 to 2013. In 2014, LSG was the most common weight reduction procedure (45.9%), overcoming RYGB [3]. Laparoscopic gastric bypass surgery (LMGB), which Rutledge first introduced, is a technology that uses vertical tube gastroplasty in combination with gastric loop bypass surgery. In 2013, several surgeons specially trained with this procedure offered denomination mini-gastric bypass/single gastric bypass anastomosis (OAGB/MGB) [1, 3]. OAGB/MGB seems very efficient in decreasing obesity-concerning comorbidities. It gives a perfect quality of life with such good sequels. Though not formally distinguished in America as a weight reduction surgery, the orientation towards MGB and OAGB in the Pacific/Asia and Europe is fast rising, putting it as the most frequent surgery following LSG [4–5]. Through the years, the expression used for surgical procedures in patients with morbid obesity was mainly “bariatric surgery”. The main ending point of these techniques is lowering BMI and body weight. However, it became apparent that the benefits and mechanisms of BS extend beyond weight loss: T2DM (type 2 diabetes mellitus) and correlated comorbidities can dramatically improve post-operation. The concept determined the expansion of a novel idea of surgery, named diabetes or metabolic surgery [2, 4]. The safety and efficacy of BS in treating DM type 2 in obese patients are recently explained in many published articles. The scientific community has bespoken BS in patients with body mass index (BMI) of more than 35 kg/m^2^ and diabetes as alternative management choice for inpatient medical regimens [5]. Diabetes/metabolic surgery parameters are used to estimate the need for the surgery to be redefined. Treatment failure and success should be evaluated by improvement or remission of T2DM and associated comorbidities, rather than simply checking weight loss [2–4]. In diabetes surgery, gain parameters should consider not only weight and BMI but mainly the HbA1c, C peptide, fasting glycemic, levels of insulin, lipid profile, and comparable indexes [4–6].

This research focused on (1) estimating the impact of LSG and MGB on loss of weight and the remission diabetes mellitus type 2 through two years of monitoring, (2) assess the differences in efficacy and priority between the two procedures, and (3) identify which one is proper for patients according to their comorbidities.

## Material and Methods

Individuals were divided into two groups according to the laparoscopic procedures undergone (SG or MGB) at Al Sader Medical city, Al Gadeer private hospitals in Najaf city Iraq, for 24 months (Jan 2016 to Feb 2018). After their bariatric surgery, patients were monitored and followed prospectively within different interval periods (3, 6, 12, 24 months). Thirty-five obese and morbidly obese patients in our research had an established history of type 2 diabetes mellitus diagnosed two years before surgery. They were treated with hypoglycemic agents (glibenclamide, metformin) and insulin. Their ages ranged from 25 to 60 years old. Their BMI ranged from 33–60 Kg/m^2^, fasting blood sugar ranged from 150 – >300 mg/dl, and HbA1c ranged from 7–14%. Fifteen patients underwent uneventful LSG (6 females and nine males). Twenty patients underwent uneventful MGB surgery (6 females and 14 males). The patients were prepared for the surgery considering a complete history, including medical history and lifestyle, which were investigated with complete blood count, liver function tests, ESR, renal test, other important hormonal analysis, lipid profile, HbA1c, and blood sugar. A physician evaluated the patients through ECG and Echocardiogram, and chest X-ray. All patients had abdominal ultrasound examinations, and a few of them had upper gastrointestinal (GI) symptoms subjected to upper endoscopic examination according to suspicion of upper GI problems. A few of them need psychological assessment. Patients with super morbid obesity were instructed to have two weeks of a dietary regime (low fat and low carbohydrate diets) to reduce liver mass for facilitating the surgery.

### Procedure and surgical technique

All patients received general anesthesia and prophylactic antibiotics before the surgery. A special surgical couch movement at least 45° of reverse Trendelenburg position with legs spread is desirable. Supine patient position on the table with extended arms. The patient should be fastened to the table to avoid movements through posture changes. Adequate padding and warping of legs with bandages and separated (mechanical prevention of DVT), surgeon stands in between the patient’s legs on the right side. Five or four ports were used. Creation of pneumo-peritoneum by veress needle. The optical port (12 mm) was inserted in the midline of the abdomen about 20 cm from the xiphisternum. The surgeon’s left-hand port in the right upper quadrant (5 mm) and right-hand port (12 mm) for the Ligasure device and stapler inserted 10 cm from the optical port in the left upper quadrant.

The liver left lobe could be retracted by attaching a traumatic Babcock to the diaphragm left crus or Nathanson’s liver retractor. In LSG, identify the pylorus: using a grasper as marked and expanding the stomach wall, a distance of 3–6 cm over the greater curvature of the stomach measured and marked to indicate where to begin the gastric resection. The greater curvature is skeletonized by division of omentum from the marked area until the angle of His by Ligasure device. Attachment of the upper pool of the spleen is dissected precisely to prevent splenic injury and bleeding. The fundus was completely mobilized with the posterior gastric wall. Bougie tube size 36 Fr was introduced carefully by the anesthesiologist and pressed down to the pylorus and with the lesser curvature to measure the resection. The resection was performed alongside the calibrated Bougie tube through the body and fundus. A linear stapler loaded with 60 mm cartridges with different heights started 4.1 mm near the antrum to 3.8 on corpus and fundus. After the leak test, the gastric specimen can be extracted through the slightly enlarged 12 mm right port. Drain was put on stapler’s lines site. In MGB surgery, lesser omentum is opened between third and fourth Lt arterial gastric vessels, then a 60 mm cartridge articulating handle cutting the stomach transversely, then a 60 mm cartridge cutting the stomach up to the angle of His over 34 Fr Bougie tube. Thus, long pouch in the lesser curvature is created accommodating around 120–150 ml. Ligament of Treitz was identified, 150–175 cm long loop of small bowel from duodenal-jejunal junction measure then anastomosed with the pouch with 60 mm cartridge, anterior to the transverse colon. In the case of heavy omentum, the omentum is divided.

A leak test is done by rinsing the stapler line with saline fluid and insufflating the stomach with air, then monitoring the air bubble or pushing 200 ml diluted blue methyl to the sleeved stomach and monitoring if there is leakage, drain put at the anastomosis site. The nasogastric tube was removed on day 0 postoperative, and patients were encouraged to move early. On the first day postoperatively, a leak test with Methylene blue was performed, and if there were no leakage signs, they could start oral intake. Usually, the patients received their discharge on the first post-operative day. We recommended starting liquid diets in the first two weeks. The patients were scheduled to submit an estimation of nutrition status, clinical evaluation, and laboratory studies at the first month and then every three months after that. After discharge, vitamin supplements were routinely prescribed. At each follow-up visit, glycemic control status and weight loss (blood sugar, HbA1c, and hypoglycemic management) were estimated. Diabetes remission (DR) means HbA1c less than 6.5 without oral hypoglycemic treatments or insulin.

### Statistical analysis

Data of the patients were entered and transformed into a computerized database with statistical utilities (SPSS) version 22, USA, used in statistical procedures. Descriptive statistics are presented as frequencies (patients), proportions (%), mean and standard deviation, and appropriate statistical tests were applied accordingly. The significance level was set at 0.05. Final findings are presented in tables or figures with an explanatory paragraph for each.

## Results

The operation time varied between 60 and 120 minutes. The postoperative stay in hospital ranged from 1–2 days. Twenty patients in the MGB group and 15 in the LSG group enrolled in this study, and no statistically meaningful differences were observed between the two groups in terms of the basic characteristics, P value>0.05 as (Table 1 and 2). We found a significant difference in BMI within studied groups from baseline levels; in the MGB group, BMI mean 46.04 kg/m^2^ reduced to 41.83 kg/m^2^ after three months then to 38.28 kg/m^2^ at six months, 30.93 kg/m^2^ at 12 months and finally reached 25.20 kg/m^2^ at 24^th^ month (Table 3). A similar trend was reported in the LSG group, where the mean BMI at baseline was 46.53 kg/m^2^ with continuous reduction at each checkpoint to reach 24.47 kg/m^2^ at the 24^th^ month. Both groups showed statistically significant changes (P value<0.001). From another perspective, the differences between both groups at each checkpoint were not statistically significant (P>0.05). The mean difference of amplitude of change between baseline BMI and that at the subsequent checkpoints were compared. The results of these comparisons are in (Table 4).

**Table 1.** Baseline characteristics of the patients.

**Variable**		**Mini gastric bypass (n=20)**		**Gastric sleeve (n=15)**		**P value**
		**No.**	**%**	**No.**	**%**	
**Age (year)**	**≤30**	5	25	4	26.6	**0.705**
	**21–30**	11	55	10	66.6	
	**31–40**	4	20	1	6.8	
Mean age±SD *	**36.3±6.7**	35.0±6.1		36.2±7.13		0.38
**Gender**	**Male**	9	60	14	70	0.537
	**Female**	6	40	6	30	

**Table 2.** Comparison of Body mass index (BMI) of patients in both studied groups at different checkpoints (within and between groups).

**Check point**	**BMI (kg/m^2^)**				**P value (between groups)**
	**Mini-gastric bypass (n=20)**		**Gastric sleeve (n=15)**		
	**Mean**	**SD**	**Mean**	**SD**	
**Baseline**	46.53	5.64	46.04	4.45	0.774
**3 months**	40.87	4.12	41.83	3.57	0.467
**6 months**	36.27	3.94	38.28	3.93	0.144
**12 months**	30.47	3.87	30.93	3.25	0.706
**24 months**	24.47	1.64	25.20	2.38	0.313
**P value (within group)**	<0.001		<0.001		

**Table 3.** Comparison of mean differences in BMI at different check points relative to baseline values (between group).

	**Difference in BMI (kg/m^2^)**				**P value**
	**Mini-gastric bypass (n=20)**		**Gastric sleeve (n=15)**		
	**Mean**	**SD**	**Mean**	**SD**	
**Baseline – 3 months**	5.66	1.66	4.22	2.34	0.045
**Baseline – 6 months**	10.26	1.42	7.78	3.03	0.005
**Baseline – 12 months**	16.06	3.74	15.12	2.94	0.261
**Baseline – 24 months**	22.06	5.23	20.84	3.30	0.137

**Table 4.** Comparison of HbA1C levels of the patients in both studied groups at different checkpoints (within and between groups).

**Check point**	**HbA1C (%)**				**P value (between groups)**
	**Mini-gastric bypass (n=20)**		**Gastric sleeve (n=15)**		
	**Mean**	**SD**	**Mean**	**SD**	
**Baseline**	9.90	1.92	9.33	1.18	0.521
**3 months**	6.58	0.41	7.23	0.59	<0.001
**6 months**	6.00	0.28	6.60	0.34	<0.001
**12 months**	5.50	0.51	6.23	0.26	<0.001
**24 months**	5.40	0.50	5.50	1.43	0.039
**P value (within group)**	<0.001		<0.001		

The mean reduction in BMI was considerably larger in the MGB group than that of gastric sleeve, 5.66 versus 4.22, respectively, (P value=0.045), at three months, the reduction in BMI was also larger, (P value=0.005). At the 12^th^ and 24^th^ months, the reduction in both groups was not significantly different (P value>0.05). Furthermore, the percentage was calculated, and it was greater in the MGB group than SG, where the percentage change at the endpoint was 47.4% in MGB compared to 45.3% in LSG (P value=0.03). Regarding the HbA1C, a similar trend was reported and changes to BMI. As shown in (Table 5 and 6).

**Table 5.** Comparison of HbA1C levels of the patients in both studied groups at different checkpoints (within and between groups).

**Check point**	**HbA1C (%)**				**P value (between groups)**
	**Mini-gastric bypass (n=20)**		**Gastric sleeve (n=15)**		
	**Mean**	**SD**	**Mean**	**SD**	
**Baseline**	9.90	1.92	9.33	1.18	0.521
**3 months**	6.58	0.41	7.23	0.59	<0.001
**6 months**	6.00	0.28	6.60	0.34	<0.001
**12 months**	5.50	0.51	6.23	0.26	<0.001
**24 months**	5.40	0.50	5.50	1.43	0.039
**P value** **(within group)**	<0.001		<0.001		

**Table 6.** Comparison of mean differences in HbA1C (%) at different check points relative to baseline values (between groups).

	**Difference in HbA1C (%)**				**P value**
	**Mini-gastric bypass (n=20)**		**Gastric sleeve (n=15)**		
	**Mean**	**SD**	**Mean**	**SD**	
**Baseline – 3 months**	3.33	1.87	2.10	0.95	0.107
**Baseline – 6 months**	3.90	2.03	2.73	1.03	0.026
**Baseline – 12 months**	4.40	2.14	3.10	1.06	0.025
**Baseline – 24 months**	4.50	2.10	3.83	1.55	0.382

A significant change was found in HbA1c levels in both studied groups from baseline levels. In the MGB group, the mean HbA1c was 9.90%, reduced to 6.85% after three months, then to 6.00% at six months, 5.5% at 12 months, and finally reached 5.40% at 24^th^ month. A similar trend was reported in the LSG group, where the mean BMI at baseline was 9.33%, with continuous reduction at each checkpoint to reach 5.50% at the 24^th^ month. Both groups showed statistically significant changes (P value<0.001). In the MGB group, there were 16 patients from 20 patients who had achieved DR in the first month after surgery, and four patients achieved DR during the first three months after surgery. In LSG, 12 patients from 15 patients achieved DR without medications in the first month after surgery. Four patients were on metformin tablets 500 mg twice a day for six months after surgery, and then they had DR from diabetes. Moreover, one patient continued on metformin tablets 500 mg twice a day, and his HbA1c was 6.5%. Furthermore, the change in BMI was observed across the age and gender in both studied groups (Table 7 and 8). This comparison revealed that the change was larger in the older age group and female gender (P-value <0.005). For the connection between the change in HbA1C level and BMI (Table 9 and 10), bivariate correlation test revealed an inverse correlation between change in BMI and LSG (R=-0.391, P=0.003), according to the value of R, the correlation was stronger in MGB.

**Table 7.** Comparison of percentage change in HbA1C levels of the patients in both studied groups at different checkpoints (between groups).

	**Percentage change in HbA1C (%)**				**P value** **(between groups)**
	**Mini-gastric bypass (n=20)**		**Gastric sleeve (n=15)**		
	**Mean**	**SD**	**Mean**	**SD**	
**Baseline – 3 months**	33.5%	8.5%	22.5%	13.0%	0.022
**Baseline – 6 months**	39.4%	8.4%	29.3%	13.0%	0.027
**Baseline – 12 months**	44.4%	7.8%	33.2%	13.9%	0.012
**Baseline – 24 months**	45.5%	16.8%	41.1%	14.0%	0.298

**Table 8.** Comparison of overall change in BMI (baseline – 24 months) according to age and gender in both studied group.

		**Change in BMI (baseline – 24 months)**		**P value**
		**Mean**	**SD**	
**Mini-gastric bypass (n=20)**				
**Age (year)**	**≤30**	21.00	2.37	0. 041
	**31–40**	21.23	3.42.	
	**>40**	23.60	4.15	
**Gender**	**Male**	20.22	2.33	0.025
	**Female**	24.83	7.25	
**Gastric sleeve (n=15)**				
**Age (year)**	**≤30**	19.50	1.29	0.010
	**31–40**	18.89	1.90	
	**>40**	24.11	3.08	
**Gender**	**Male**	20.49	3.49	0.035
	**Female**	22.67	2.94	

**Table 9.** Comparison of overall change in HbA1C (baseline – 24 months) according to age and gender.

		**Change in HbA1C (baseline – 24 months)**		**P value**
		**Mean**	**SD**	
**Mini-gastric bypass (n=20)**				
**Age (year)**	**≤30**	2.88	0.63	0.327
	**31–40**	4.10	1.73	
	**>40**	5.12	1.3	
**Gender**	**Male**	3.83	0.94	1.00
	**Female**	3.89	0.88	
**Gastric sleeve (n=15)**				
**Age (year)**	**≤30**	2.25	1.50	0.034
	**31–40**	4.67	1.58	
	**>40**	5.57	2.30	
**Gender**	**Male**	4.57	2.41	0.827
	**Female**	4.33	1.51	

**Table 10.** Correlation of change in BMI and HbA1C (baseline to 18 months) with age and gender of the patients.

	**Group**	
**Correlation between Change in BMI and HbA1C**	**Mini-gastric bypass**	**Sleeve group**
**Correlation coefficient**	-0.49	-0.391
**P value**	0.002	0.003

## Discussion

In our time, obesity is considered a great public health interest. Epidemiological studies showed that obesity affects more than 400 million adults in the world [1]. There are difficulties in its management, with different treatment lines used, including dietary restriction, behavioral approaches, regular aerobic exercises, and pharmacological agents. All these approaches result in a moderate benefit [2]. Furthermore, the problem is gaining additional weight. When medical therapy fails, surgical approaches in bariatric operations are chosen for severe and resistant patients [3, 4]. Insulin resistance is mainly caused by obesity-induced DM type 2 (T2DM), and both conditions are termed “diabesity”. The prevalence of T2DM worldwide is about 300 million people, and more than 60% of them are obese. Thus, the treatment and prevention of such conditions are vital for improving public health [5]. Previously published articles concluded that surgical intervention in bariatric surgery efficiently produced the target loss of weight with the resolve of obesity-related complications in patients [6–7]. In selected obese patients with T2DM, bariatric surgery was efficient management for T2DM compared with medical management [8].

In the current years, many clinical trials have considered the specific pre-operative parameters that may be considered predictors, thereby permitting surgeons to select the most suitable patients who may benefit from surgical intervention for best glycemic control. The main preoperative predicting factors for diabetic control are age, BMI, C-peptide, gender, diabetes duration, glycemic control, insulin use, and surgical procedure type. Rutledge *et al.* declare body weight loss percentage excess (%EWL) of 77% in 2 years in 1,274 patients case series [9]. Carbajo *et al.* recorded a %EWL of 80% in 2 years, deeming a series of 209 patients [10]. In a previous literature review, Quan *et al.* compared OAGB with laparoscopic adjustable gastric banding (LAGB), laparoscopic SG (LSG), and laparoscopic RYGB (LRYGB). OAGB demonstrated significantly higher weight reduction in contrast to LAGB, LSG, and LRYGB [11]. Weight loss outcomes are very good with LSG surgery in the short-term and long-term outcomes. According to the 2012 Fourth International Consensus Summit, accounting for 46,133 LSG done by 130 surgeons worldwide, the percentage of weight loss (%EWL) at 1 and 2 years was 59.3%, 59.0%, respectively. Hormonal changes caused by excision of the stomach or part of it may be the underlying mechanism behind diabetic control following gastric bypass procedures. Cummings explained these hormonal changes in 2009 and reviewed the raised hypotheses related to the mechanisms underlying DR.

Three hypotheses compromise the ghrelin hypothesis, the upper and the lower gastrointestinal hypotheses. The ghrelin hypothesis established on a review [12] supposed that RYGB causes disturbance in the regulation of the ghrelin hormone. The hormone Ghrelin is released by the proximal small intestine and stomach, specifically pre-meals. Its main physiological actions contain a raise in fat mass raise and appetite [13]. In favor of the ghrelin hypothesis, many studies have shown that ghrelin hormone levels are very low after RYGB. Decreased release of ghrelin may reduce food consumption, decrease appetite and have a function in raising glucose tolerance, as ghrelin can stop counter-regulatory hormones [14]. The lower intestinal hypothesis supposes that intestinal shortcuts following BS raise the flow of nutrients ingested and raise secretion of glucagon-type peptide-1 (GLP-1). GLP-1 is an incretin hormone that is released by endocrine L cells as peptide. These cells are widely spread through the small bowel and at an elevated thickness of the ileum. GLP-1 stimulates insulin release and has also been shown to decrease proliferation and suppress apoptosis of beta-cells [12, 13]. Both BPD and RYGB result in alimentary tract shortcuts, and published articles have shown that post food intake, GLP-1 release is elevated after surgery [15]. Therefore, it seems logical that the secretion of GLP-1 may be stimulated after surgical operation, leading to more secretion of insulin. This theory could also clarify the expansion in β-cell mass and hyperinsulinemia hypoglycemia that follows RYGB surgery [16]. The upper gastrointestinal hypothesis states that loss of nutrients in touch with the duodenum is the main factor in improving glycemic control. The basic concept of this hypothesis is that factors of unknown origin or certain processes in the duodenum induce glucose homeostasis. Rubino *et al.* [17] were the first to explain this hypothesis. They acted on a variant of RYGB surgery to create the intestinal bypass and let the stomach unaffected, which encouraged the same digestive discontinuation without re-anastomosis. This type of bariatric surgery is called duodenal-jejunal bypass and was tested in several clinical trials, showing an improvement in DM type2 without reducing body weight [11]. These studies finally suggested that LSG and MGB have novel and efficient weight reduction types of BS [12].

There is growing proof indicating that SG significantly improves glycemic control in most morbidly obese patients with T2DM [9–12]. In a previously published systematic review, the percentage of diabetic resolution following SG surgery ranges from 80–96% in morbid obese patients. A similar scope was following RYGBP in diabetic patients [15]. Moreover, laparoscopic surgery MGB type is considered a safe alternative to LRYGB surgery, demonstrating the same efficacy in weight loss, and improving metabolic complications, like diabetes [11–14]. Follow-up of patients in both short and long-term periods assure the beneficial impact of these simple techniques for morbidly obese and obese subjects with T2DM [16]. Laparoscopic MGB surgery in morbidly obese subjects with T2DM must be efficient in prospective randomized trials and comprehensive reports in the published literature [14–16]. Disturbance of ghrelin levels happened after the LSG surgery. LSG is also considered to have a hindgut influence with raising GLP-1 and peptide Y levels due to a rise in transit time after the operation [8–10]. As gastric sleeve pouch construction is the primary step of this operation, similar techniques are engaged in MGB surgery. The only anatomical area that was not affected by LSG was the foregut. Specifically, the upper bowel segment was not in touch with ingested nutrients in the GB-treated group compared to the SG-treated group where made contact with him. In a recent study, Lee *et al.* [14] were the first to compare the efficacy of LSG and MGB on diabetic control. The results exhibited strong evidence that supported the hypothesis that the exclusion of duodenum may have some role in diabetes mellitus control post bariatric surgery in obese patients.

Our outcome supported the surveillance of Lee *et al.* to morbidly obese patients. Despite the difference in the patient population included in this study, our outcome also emphasizes that MGB is accompanied by better glycemic control and a higher DR. In combination with the evidence that weight reduction is the same after LSG and MGB techniques, these results still support Lee *et al.*’s proposed hypothesis [14]. Thus, the role of weight loss and the hindgut and ghrelin theories are excluded. The only remaining explanation of the different results is the foregut (i.e., duodenal exclusion). In other words, the mechanism indicates potential superiority of the MGB over the LSG in achieving diabetes resolution, but more data are required to build up this conclusion. However, previous evidence assures that the gold standard for DR is the Roux-en-Y gastric bypass. Due to identical mechanisms, DR may also be engaged to facilitate this procedure.

The MGB could be a reasonable alternative. Even though our findings appear to promote and support MGB as an effective DR treatment strategy, additional research is required to supply final findings on the perfect process for obtaining DR [15]. Diabetic control and resolution are regarded as the percent weight loss instead of basic weight. Body mass index (BMI) was extensively studied as a predicting parameter for DM type 2 after bariatric surgery. Mingrone *et al.* suggested that the baseline BMI was not related to DR in morbidly obese subjects [16]. They reported similar DR rates in patients with a BMI of more than 35 kg/m^2^ versus a BMI of less than 35 kg/m^2^ [17]. WJ Lee *et al.*, in institutional research, also present the same DR rates in obese patients with a BMI of 30 kg/m^2^ against a BMI of less than 30 kg/m^2^ [18]. Several studies considered BMI as a predictor of DR type 2. Lakdawala *et al.* found that BMI equal to or less than 35 kg/m^2^ is considered as predicting factor for long-term DR [19]. In a study conducted on the Korean population, Lebovitz *et al.* found that BMI ≥35 kg/m^2^ is a predictor for long-term DR [18]. Park JY. demonstrated that in the first year, a BMI less than 35 was a positive predicting factor of DR [19]. On the other hand, a BMI >50 kg/m^2^ was an adverse predicting factor of DR type 2 [20]. Our study’s correlation between BMI and Diabetes type 2 is fairly significant and precisely with GS more than GMBS surgery. The statistical significance of our study regarding the overall changes of BMI and HbA1c according to age and gender occurred because of the relatively small sample size. The ages of almost all patients were between 35 and 45-year-old. Furthermore, our outcome should be evaluated in larger studies.

## Conclusions

Both surgeries were helpful and had valuable results in patients. Still, the gastric mini-bypass procedure was more efficient than sleeve gastrectomy in controlling the remission of type 2 DM without the need for medications on short term follow-up of this study. Gastric sleeve surgery and gastric mini bypass surgery decrease body weight and control and remission of type 2 DM. There should be long-term monitoring and concentrated studies to reveal the effects and benefits to patients within longer periods after surgery.

## Acknowledgments

### Conflict of interest

The authors declare that there is no conflict of interest.

### Ethics approval

This study was approved by the ethical committee of the Faculty of Medicine, Jabir Ibn Hayyan Medical University (315 JMU-6^th^ Feb 2019).

### Consent to participate

All patients received informed consent forms before participating in the study.

### Authorship

SMMAH collected data, designed the concept of the manuscript, did the writing, analysis, and results of the manuscript, submitted the manuscript, revision and gallery proof. MAZA participated in data collection and recording results. AAAW and ASM took part in data collection. SSAB took part in data collection and data analysis.
